# Enhancement of Proton Conductivity in Perfluorosulfonic Acid Membranes with the Addition of Hydrophilic Fillers

**DOI:** 10.1021/acsomega.6c00896

**Published:** 2026-08-04

**Authors:** Tatsuki Yasuda, Akihito Yamano, Ryuji Ohno, Katsuyoshi Kakinuma

**Affiliations:** † Integrated Graduate School of Medicine, Engineering, and Agricultural Sciences, 38146 University of Yamanashi, Takeda 4, Kofu, Yamanashi 400-8511, Japan; ‡ Hydrogen and Fuel Cell Nanomaterials Center, University of Yamanashi, Miyamae 6-43, Kofu 400-0021, Japan; § Clean Energy Research Center, University of Yamanashi, Takeda 4, Kofu 400-8511, Japan

## Abstract

The present work demonstrates an improvement in proton conductivity at 80 °C as a result of the addition of a hydrophilic filler (carboxymethyl cellulose nanofibers, CMCNFs) to Nafion composite membranes. The composite membranes (CMCNF/Nafion) prepared by die coating are translucent and flexible, without pores or cracks. The proton conductivity of the CMCNF/Nafion membrane reaches 0.165 S cm^–1^ at 80 °C and 80% RH. The water retention of the membranes, based on DSC measurements, indicates that the amount of nonfreezing water (water that does not freeze down to −55 °C) is independent of the addition of CMCNF, but the amount of capillary water (water that melts between −55 and 0 °C) increases with CMCNF addition. We considered that the presence of capillary water at the interface between CMCNFs and Nafion would be one of the essential factors that improve the proton conduction in the electrolyte. The small-angle X-ray scattering (SAXS) profiles prove that the size of the hydrophobic domain water clusters in Nafion becomes smaller with the addition of CMCNF, while the water content remains the same. The microstructure of the CMCNF/Nafion composite membrane and the behavior of the water content (capillary water) would be one possible contribution to show the improvement of proton conductivity.

## Graphical abstract

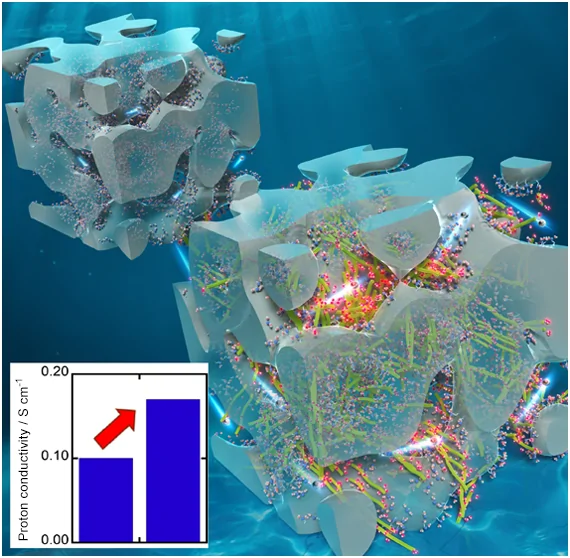

## 1 Introduction

Light-duty and heavy-duty vehicles (LDVs and HDVs, respectively) equipped with internal combustion engines emitted 3.8 Gt of CO_2_ globally in 2023.^1^ To achieve carbon neutrality, these systems should be replaced by electric vehicles. Typical HDV transportation systems require high mileage, usually greater than 500 miles per day, and thus must be equipped with the ability to carry the equivalent of up to ca. 1.2 MWh of electrical energy resources, which could weigh as much as 8 t or as much as one-third of the total carrying capacity. In contrast, a long-haul HDV utilizing polymer electrolyte fuel cells (PEFCs) would only require approximately 70 kg of hydrogen for a 500-mile journey and high-temperature operation, such as uphill climbing at low velocity.^2–4^ If green hydrogen is used, PEFCs are particularly suitable for HDVs. However, increased efficiency of power generation by PEFCs is also required for HDVs, with one of the most direct approaches being to improve the proton conductivity of the electrolyte membrane. Aromatic polymer membranes^5–8^ phosphonic acid-based polymer membrane,^9–11^ phytic acid-based polymer membrane,^12,13^ polybenzimidazole-based polymer membrane,^14^ and hydrocarbon-based polymer membranes^15,16^ decrease the resistive loss (overpotential) specifically by the enhancement of proton transport over the required wide temperature range. The proton conductivity of a PFSA membrane occurs via the Grotthuss mechanism or the vehicular mechanism and depends upon the water activity at each temperature. PFSA membranes also show the increase of proton conductivity by mixing the fillers, such as graphitized carbon,^17^ TiO_2_ nanoparticles,^18–21^ surface-modified SiO_2_,^22–24^ and submicron-sized or mesoporous ZrO_2_.^25,26^ These fillers have a hydrophilic surface and thus not only retain water but also have the capability of supplying additional proton-conducting pathways at the interface between the hydrophilic surface of the oxide and the PFSA membrane.^27^ Cellulose nanofibers also have hydrophilic surfaces and are promising candidate additives to improve proton conductivity.^28–37^ Yang et al. formed films using cellulose modified with 3-aminopropyltriethoxysilane (APTES), with the proton conductivity reaching 62.2 mS cm^–1^ (95 °C, 100% RH).^28^ Phan-Huynh et al. applied sulfonated cellulose in the PFSA composites to enhance the proton conductivity up to 1.79–2.98 mS cm^–1^ (20–80 °C).^30^ Guccini et al. added carboxylate cellulose nanofibers in thin membranes (15 μm) to reach proton conductivities up to 1 mS cm^–1^ (30 °C, 65–95% RH).^36^ These studies reported enhancements of the water uptake, but the proton conductivity did not exceed that of the pristine PFSA membrane. The cellulose nanofibers tend to decompose to glucose via hydrolysis under acid conditions, which might be one of the issues impeding the use of these materials as additives in PFSA composites. In the present study, we focus on the use of hydrophilic carboxymethyl cellulose nanofibers (CMCNFs), which have stability particularly at low-pH conditions, to supply the additional proton-conducting pathways with higher water uptake over a wide temperature range. We investigate the proton conductivity, microstructure, and water uptake of PFSA membranes with CMCNFs as additives toward the application of these membranes in PEFCs operating even at elevated temperatures.

## 2 Experimental Section

The CMCNF-dispersed solvent (2 wt %, average fiber diameter 10 nm, Sugino Machine Co., Ltd.) and Nafion-dispersed one (20 wt %, DE2020 CS, Chemours Co.) and potassium hydroxide (86%, Kanto Chemical Co., Ltd.) were selected as starting materials. A mixture of 50 mL of Nafion dispersion and 0.80 g of potassium hydroxide was stirred for 3 days using a stirrer (RSH-4DN, AS ONE Co., Ltd.). Hereafter, the obtained dispersion solution is denoted as K-Nafion (pH = 4.02). CMCNF solutions (6, 10, 14, 20 mL), ethanol (0.20 g), 1-propanol (1.0 g), and 2-propanol (0.020 g) were mixed for 1 h using a rotating ultrasonic dispersing device (PR-1, SHINKI Co., Ltd., 400 rpm). Subsequently, the slurry and K-Nafion dispersion were mixed using an ultrasonic dispersing device (first treatment, 400 rpm for 20 min, 3 times; second treatment, 400 rpm for 1 h, one time) and degassed for 5 min using a rotation revolution stirring defoaming machine (HM-400W, Kyoritsu Seiki Co., Ltd.). The obtained slurry was coated on a Teflon sheet using the die coating method (Mini-50, Daimon Co., Ltd.) and dried using a hot-air dryer to produce a composite membrane (9 cm × 9 cm^2^). The obtained membranes were annealed by hot pressing at 200 °C for 3 min (T1CMD-2.5, Toho Kogyo Co., Ltd.). The obtained electrolyte membranes were immersed in a 0.1 M sulfuric acid solution (80 °C) for 1 h to exchange potassium ions in the membrane with protons and then immersed in ultrapure water (80 °C) for 1 h to remove residual sulfuric acid from the membrane surface. The treated membranes were dried for 30 min in a vacuum constant-temperature dryer (60 °C, atmospheric pressure) to obtain the CMCNF composite membranes (CMCNF/Nafion).

The thickness and flatness of the CMCNF/Nafion electrolyte membranes were evaluated by using scanning electron microscopy (SEM, SU9000, Hitachi High-Tech Co., Ltd.). The distribution of CMCNFs in the membrane was characterized by transmission electron microscopy (TEM, H9500, Hitachi High-Tech Co., Ltd.). In situ small-angle X-ray scattering measurement (SAXS, NANO-Viewer, Cu­(Kα) X-ray (λ, wavelength 1.5406 Å, X-ray diameter 70 μm), Rigaku Co., Ltd.) was performed to analyze the microstructure of the electrolyte membrane at 80 °C and 20–80% RH. The scattering vector, *q*, was calculated using the Bragg equation (eq 1)­

1 $$q=\left|q\right|=\frac{4\pi }{\lambda }\sin\theta$$

Differential scanning calorimetry (DSC Q100, TA Instruments) was perfornamed to investigate the water content of the CMCNF/Nafion composite membranes in detail. The measurements were performed over a temperature range of −55 to 0 °C with a heating rate of 0.3 °C min^–1^. From the DSC curve of the heating process, the amount of bulk water (defined as water that melts between 0 and 5 °C, *W*_f_) was estimated using eq 2. The amount of capillary water (defined as water that melts between −55 and 0 °C, *W*_fc_) was estimated using eq 3. The amount of nonfreezing water (defined as water that does not freeze down to −55 °C, *W*_nf_) was estimated by subtracting the amount of *W*_f_ and *W*_fc_ from the total water content (*W*_t_) using eq 4.

2 $$W_{f}=\int_{T_{0}}^{>T_{0}}\frac{\frac{dq}{dt}}{m\Delta T_{0}}dt$$

3 $$W_{\mathrm{fc}}=\int_{<T_{0}}^{T_{0}}\frac{\frac{dq}{dt}}{m\Delta H\left(T\right)}dt$$

4 $$W_{\mathrm{nf}}=W_{t}-W_{f}-W_{\mathrm{fc}}$$

where *m* is the dry weight of the sample, d*q*/d*t* is the heat flux signal of the DSC, and Δ*H*_0_ is the melting enthalpy at the melting point (*T*_0_) of bulk water.

The proton conductivities of the prepared CMCNF/Nafion electrolyte membranes were measured by using the AC four-probe method (BEL-FCE-LT, Nippon Bell Co., Ltd.). The temperature was fixed at 80 °C, and the resistance value (*Z*) was measured using the AC four-terminal method while varying the relative humidity between 20 and 80% RH. The proton conductivity (σ) was calculated using the following equation (eq 5).

5 $$\sigma =\frac{L}{Z}\times \frac{1}{A}$$

where *L* is the probe-to-probe distance (cm) and *A* is the sample cross-sectional area (cm^2^). The thickness was calculated using the values obtained by SEM. The mechanical strengths of the CMCNF/Nafion electrolyte membrane were measured using a tensile testing machine (AGS-J 500N, Shimadzu Co., Ltd.). Dumbbell-formed electrolyte membranes with a measurement section width of 2 mm and a gripping width of 12 mm were prepared. After they reached a state of equilibrium at 80 °C and 60% RH for 2 h, tensile tests were conducted to obtain stress–strain curves. The tensile speed was set to 10 mm min^–1^.

## 3 Results and Discussion

### 3.1 Observation of CMCNF/Nafion Composite Membrane

The CMCNF/Nafion composite membranes (CMCNF content 3–10 wt %) were homogeneous and translucent without any cracks (Figure 1). The thicknesses of the CMCNF/Nafion composite membranes were ca. 23.5 μm, estimated by the cross-sectional SEM image (Figure 2), which was similar to that of a commercial Nafion membrane (NRE211, thickness 25 μm). The TEM images of the CMCNF/Nafion composite membranes showed a high state of dispersion of the needle-like aggregated CMCNFs in the membranes to obtain the adhered interface between the aggregated CMCNF and Nafion (Figure 3).

**Figure 1:**
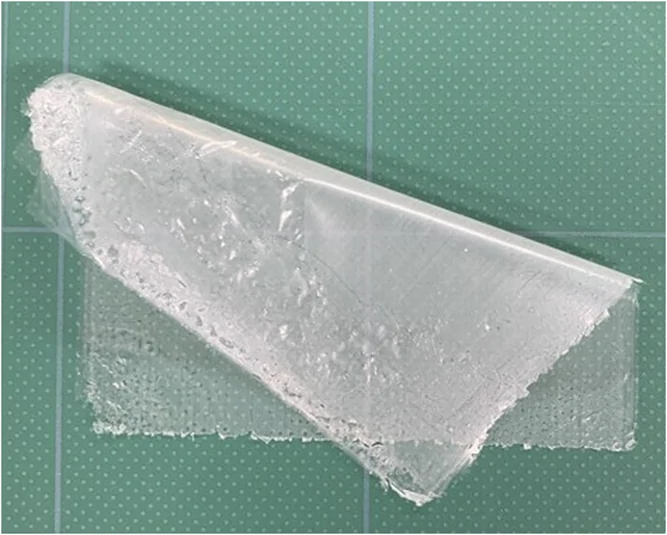
Photographic image of a 5 wt % CMCNF/Nafion electrolyte membrane [9 cm × 9 cm in size and 23.5 μm^t^ in thickness].

**Figure 2:**
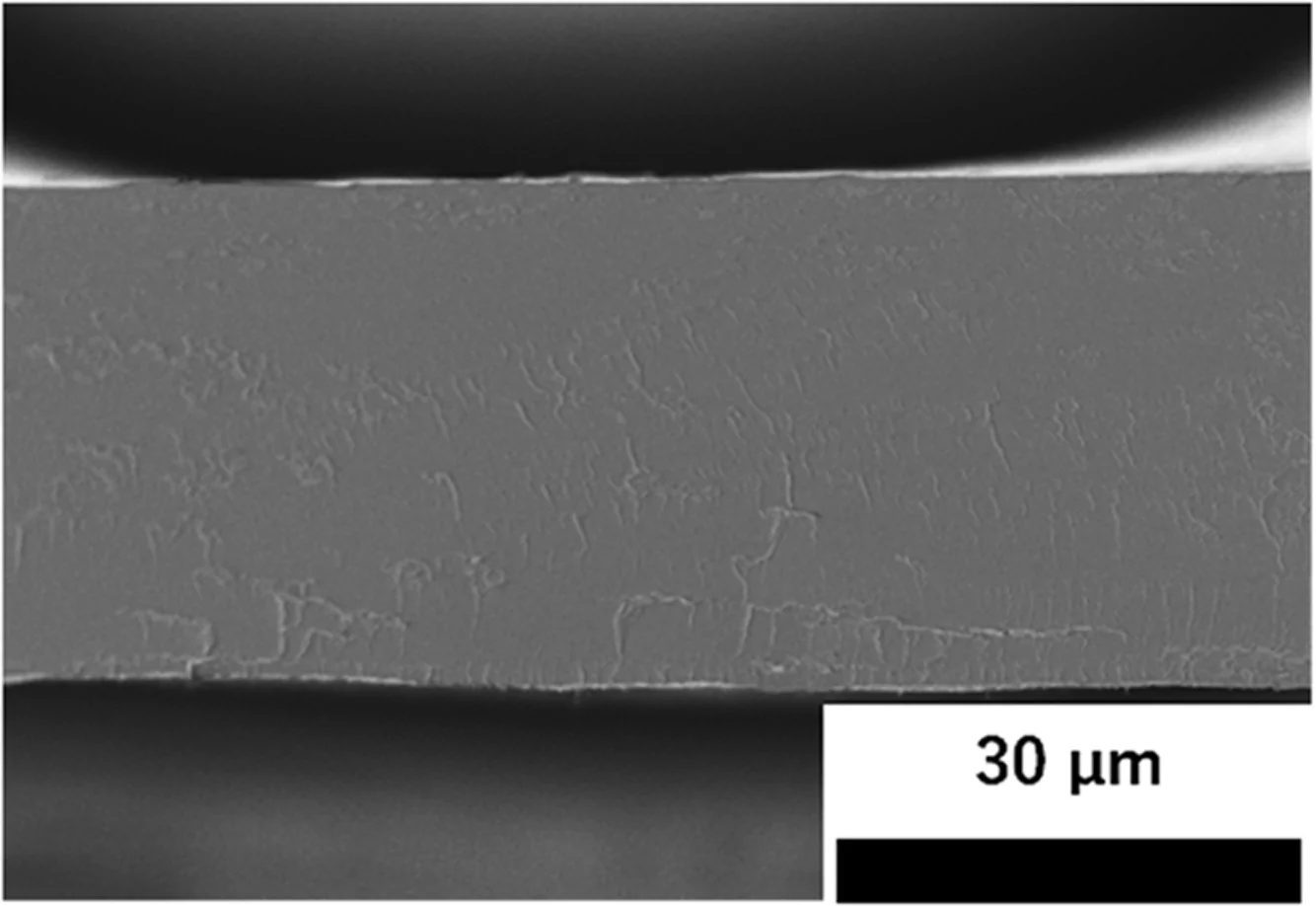
SEM image of the cross-section of a 5 wt % CMCNF/Nafion electrolyte membrane.

**Figure 3:**
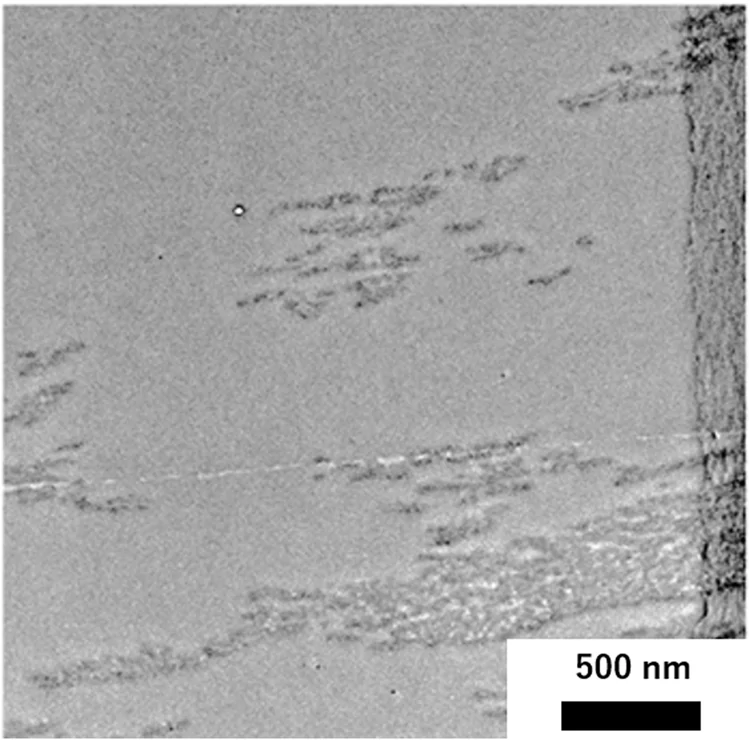
TEM image of a 5 wt % CMCNF/Nafion electrolyte membrane. The gray needle-like features are aggregated CMCNFs.

### 3.2 Proton Conductivity of CMCNF/Nafion Composite Membranes

The proton conductivities of the CMCNF/Nafion composite membranes (CMCNF content, 3, 5, 7, 10 wt %), measured by the AC four-probe method (80 °C, 80 and 20% RH), increased with the increasing CMCNF content, reaching a maximum value at 5 wt % (0.165 S cm^–1^ @ 80 °C, 80% RH), which was higher than the NEDO target value (0.12 S cm^–1^ @ 80 °C, 80% RH)^38^ and that of commercial Nafion (Figure 4a). Proton-conducting electrolyte using a cellulose nanofiber or nanocrystal only was less than 0.1 S cm^–1^ at 100% RH.^39,40^

**Figure 4:**
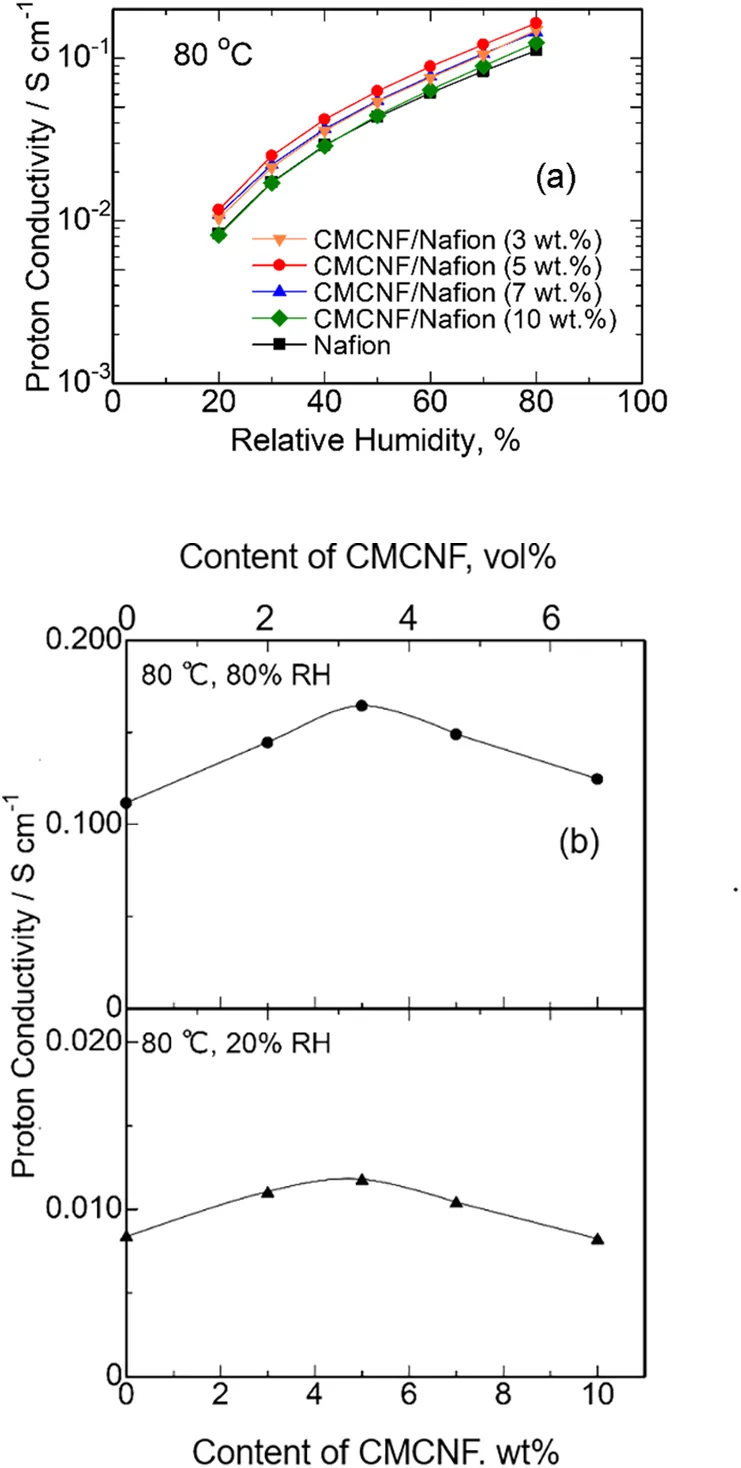
(a) CMCNF content dependence at 80 °C and 80 and 20% RH and (b) proton conductivity of 3, 5, 7, 10 wt % CMCNF/Nafion electrolyte membranes and Nafion membrane at 80 °C.

The Arrhenius plot of the CMCNF/Nafion composite (5 wt %) is given in Figure 5a. The Arrhenius plot of Nafion 211 (thickness 25 μm) was also shown as a reference. Both Arrhenius plots showed a straight line in the temperature range from 70 to 100 °C. Noteworthy, over 100 °C, the Arrhenius plot of the CMCNF/Nafion composite (5 wt %) bent slightly toward the higher-proton conduction, although the plot of Nafion remained straight at low temperature. As shown in Figure 5b, the proton conductivity of the CMCNF/Nafion composite (5 wt %) at 120 °C as a function of humidity improved further than that of Nafion. To confirm the mechanism of proton conductivity improvement, in situ SAXS and Raman are needed to analyze the CMCNF/Nafion composite (5 wt %) at present. The proton conductivities at 80 °C and 80 and 20% RH were stable for over 30 min (Figure S1), demonstrating chemical stability. We considered that the CMCNF/Nafion composite membrane has the potential to show high proton conductivity under a wide range of temperatures and humidity conditions.

**Figure 5:**
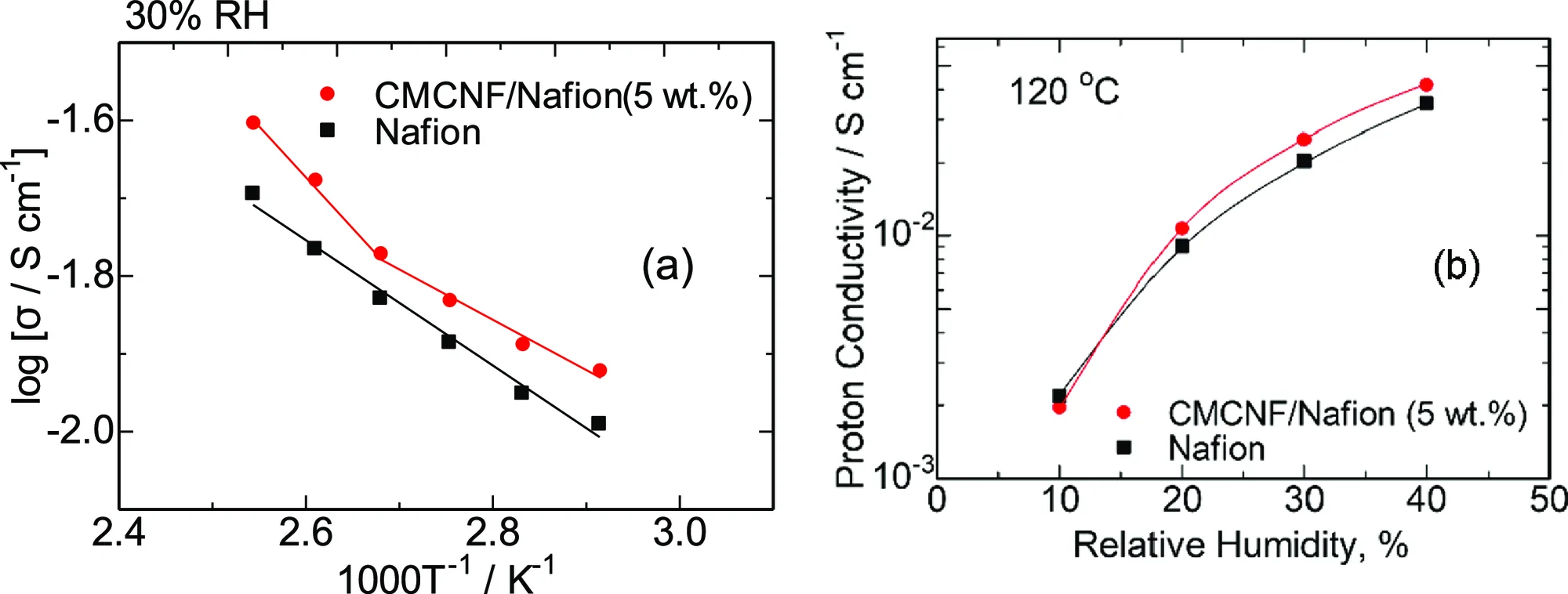
(a) Arrhenius plots of proton conductivity at 30% RH. (b) Proton conductivity of the 5 wt % CMCNF/Nafion electrolyte membrane and Nafion membrane at 120 °C as a function of relative humidity.

### 3.3 SAXS Profile of CMCNF/Nafion Composite Membranes

The in situ SAXS profiles of a CMCNF/Nafion composite membrane (5 wt % CMCNF) and Nafion are shown in Figure 6a,b, respectively. The peaks for Nafion around *d* = 10 nm (*q* = 0.6 nm^–1^), originating from lamellar crystals,^40,44^ did not shift with changing humidity. The peaks for Nafion at *d* = 3–4 nm (*q* = 1.6–2 nm^–1^), originating from hydrophilic clusters,^44–47^ shifted to higher d values with increasing humidity. This observation indicates that the incorporated water in Nafion is not associated with the lamellar crystals but rather with hydrophilic clusters. The peak originating from hydrophilic clusters in the CMCNF/Nafion composite membrane at *d* = 2–5 nm (*q* = 1.1–2.1 nm^–1^) showed similar peak intensity to that of pristine Nafion. The increased area and broader full width at half-maximum (FWHM) in Figure 6c may suggest changes in the number and/or size of clusters. However, the water adsorption measurement (Supporting Data Figure S2) showed that the water uptake of the CMCNF/Nafion composite membrane was larger than that of Nafion. These observations (SAXS and water adsorption) indicate an increase in the number of clusters and that the number of cluster *d* = 2–5 nm in the CMCNF/Nafion composite membrane would increase rather than that of Nafion (Figure 6c,d). The increase in the peak area or peak broadening occurs due to some factors, such as an increase in the number, surface cluster, aggregation, and size of particles and clusters. We need to evaluate the structures of the CMCNF/Nafion composite membrane further to understand the mechanism of proton conductivity. At present, it is supposed that the increase in the number of hydrophilic clusters would be one of the reasons for proton conductivity.

**Figure 6:**
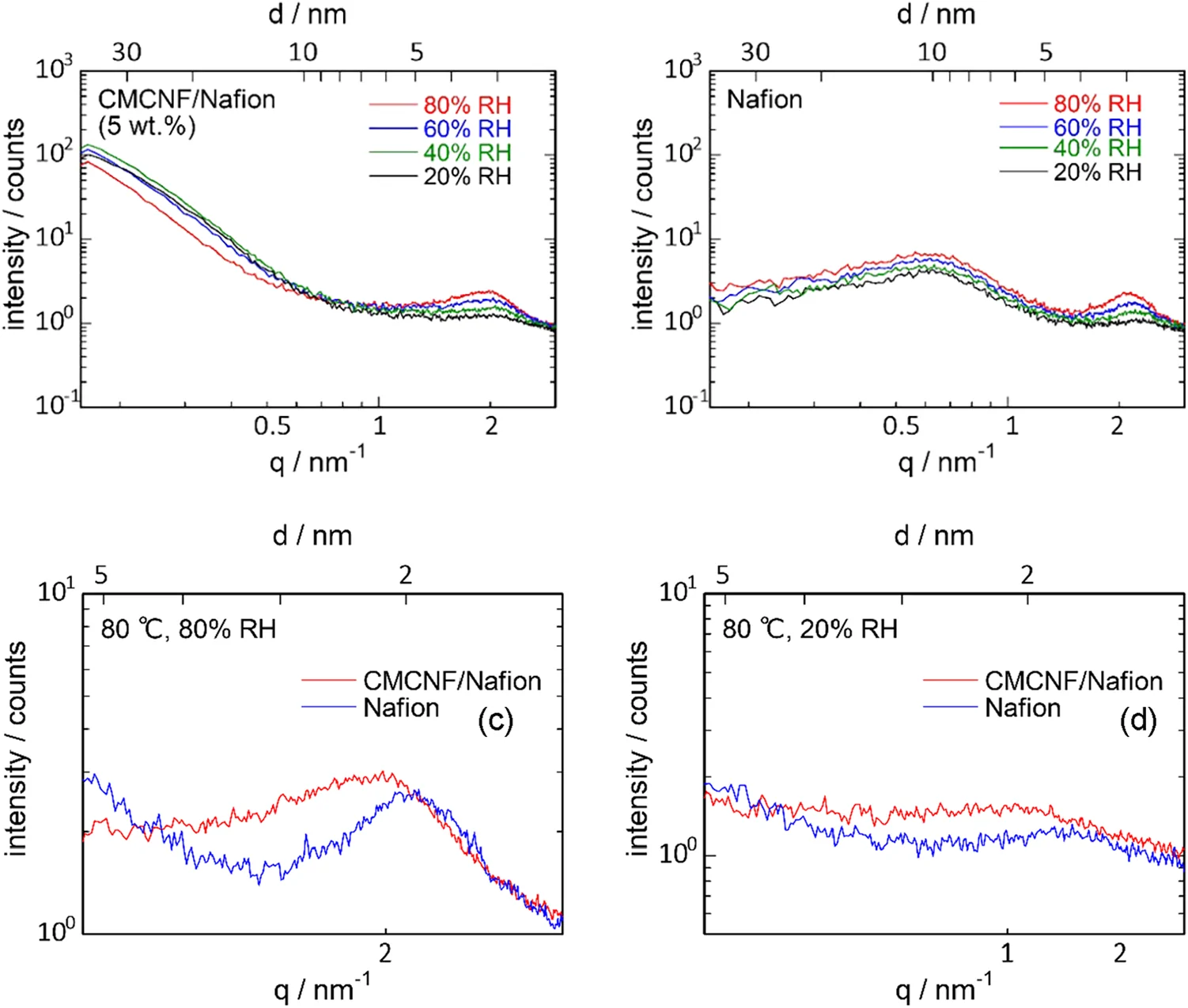
In situ SAXS profiles of (a) CMCNF/Nafion composite membrane, (b) Nafion membrane, (c) 80 °C, 80% RH, and (d) 80 °C, 20% RH.

### 3.4 Water Content of CMCNF/Nafion Composite Membranes

The *W*_f_, *W*_fc_, and W_nf_ values (bulk, capillary, and nonfreezing, respectively) for each membrane estimated from the DSC measurements are summarized in Figure 7. The *W*_f_, *W*_fc_, and *W*_n_ have different phase transition temperatures and transition heat capacities. In this study, the temperature scanning rate of the DSC measurement was 0.3 deg·min^–1^ to detect the transition heat transfer of each water. The values for pristine Nafion are *W*_f_ (17%), *W*_fc_ (9%), and *W*_nf_ (18%). The *W*_nf_ values for the CMCNF/Nafion composite membranes did not vary significantly with CMCNF content, being similar to that for the Nafion membrane. The *W*_fc_ values increased slowly up to 3 wt % of CMCNF and then increased sharply to a maximum at 5 wt % of CMCNF and then decreased slightly. The *W*_f_ values for each CMCNF/Nafion composite membrane were smaller than those of pristine Nafion. The interface between Nafion and CMCNF is one of the restricted limiting areas by adsorbed molecules and defects, where the capillary water (freezes under the temperatures between −55 and 0 °C) would increase.^41–43^ The clarification of the relationship between proton conductivity and capillary water needs further multimodal analysis from the viewpoints of a lot of factors for proton conductivity, such as polymer structure, microstructure, and other properties. It is supposed that the phenomena of capillary water would be one of the reasons for the proton conductivity enhancement at the composite membrane.

**Figure 7:**
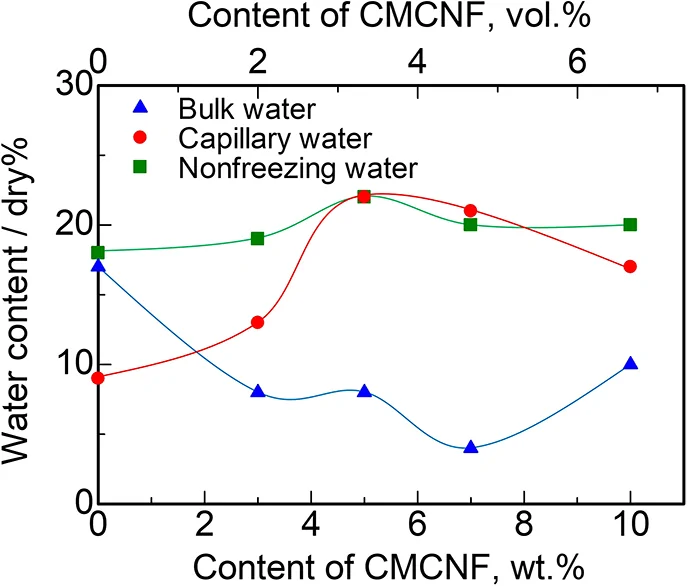
DSC measurement of the 5 wt % CMCNF/Nafion composite membrane and Nafion.

### 3.5 Mechanical Strength of CMCNF/Nafion Composite Membranes

Young’s modulus, maximum stress, and strain at break values of the CMCNF/Nafion electrolyte membranes were evaluated by means of the tensile test. Representative stress–strain curves of the CMCNF/Nafion electrolyte membrane at 80 °C and 60% RH are shown in Figure 8a. The Young’s moduli and maximum stress of each membrane are estimated from Figure 8a and are shown as functions of the CMCNF content (Figure 8b). The stress–strain curves exhibited biphasic behavior characterized by an initial linear stress–strain, followed by yielding by a second horizontal phase until fracture for all CMCNF/Nafion membranes (Figure 8a). This biphasic behavior has been widely observed in Nafion composites and cellulose-based composites.^31,48^ The Young̀s modulus and the maximum stress increased with increasing CMCNFs content up to 5 wt %, and then decreased, where the aggregation of CMCNF occurred as discussed in Section 3.2. Each CMCNF/Nafion membrane elongation also changed to a higher strain to break with the increasing CMCNF content up to 5 wt % and then decreased. The reinforcement induced by the interaction between functional groups of OH on nanoparticle filler surface and polar groups of membrane enhances the mechanical strength for these composite membranes.^49,50^ We need further experiments to clarify the mechanical strength of CMCNF/Nafion composite membranes, but the improvement by adding the CMCNF is an essential result, which should be evaluated in structure and interaction of the composite membrane.

**Figure 8:**
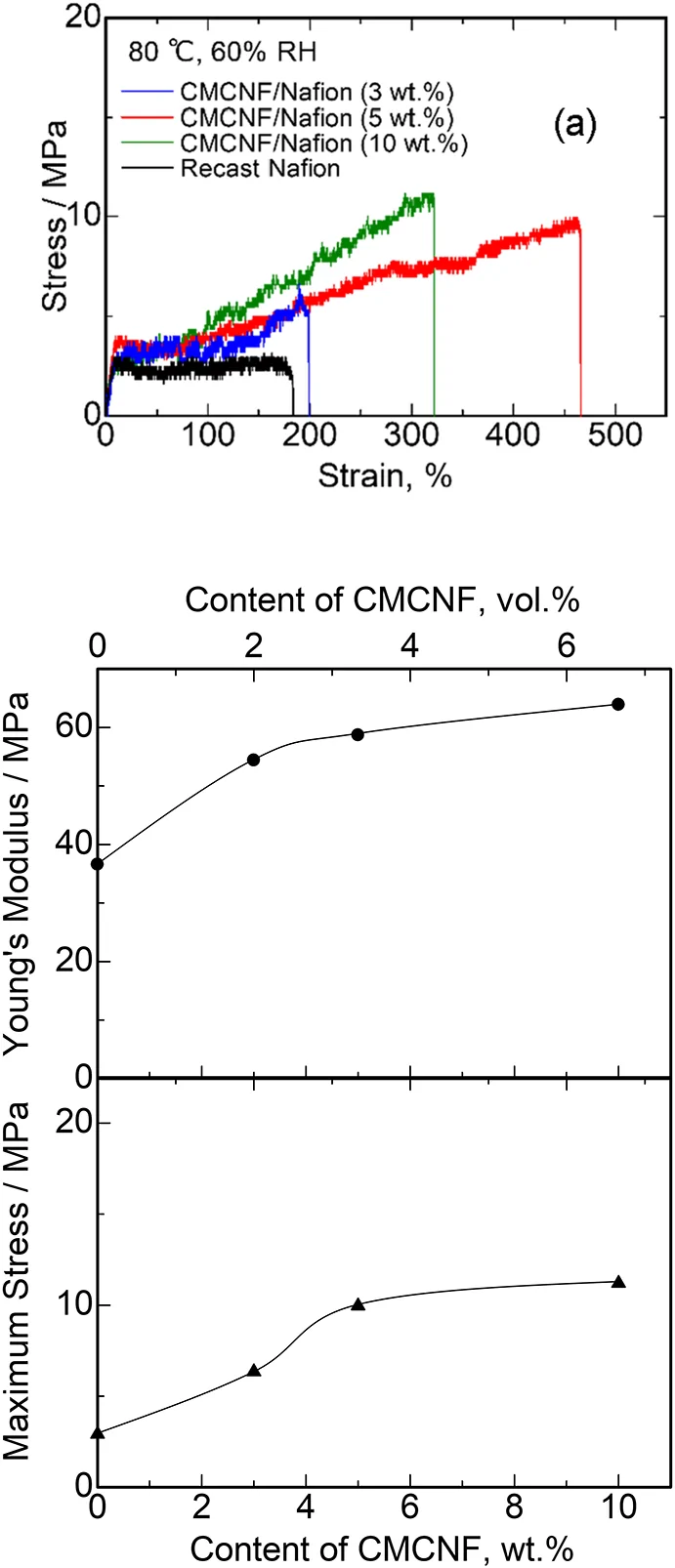
(a) S–S curve at 80 °C, 60% RH of the CMCNF/Nafion membrane and recast Nafion membrane. (b) Young’s modulus and maximum stress as a function of CMCNF content.

## 4 Conclusions

In the present study, we have fabricated a series of composite membranes (CMCNF/Nafion electrolyte membrane) by adding CMCNFs with high hydrophilicity and strength into Nafion. Slightly aggregated CMCNFs were confirmed to be dispersed within Nafion and did not form voids at the interfaces with Nafion. The proton conductivity of the CMCNF/Nafion electrolyte membranes at 80 °C reached 0.165 S cm^–1^ at a CMCNF content of 5 wt %, a value exceeding the NEDO 2030 target value (0.12 S cm^–1^ @ 80 °C, 80% RH). In SAXS measurements, the peak originating from the hydrophilic clusters in the CMCNF/Nafion electrolyte membrane exhibited relatively higher intensity than that in Nafion. From the microstructure of the CMCNF/Nafion composite membrane and the behavior of both water content and capillary water, the capillary water at the interface between Nafion and CMCNFs would be one possible contribution to improving proton conductivity.

## Supplementary Material



## Notes

Five weight percent CMCNF/Nafion electrolyte membrane and steady-state proton conductivity (PDF)
