# Supplementary material for: Enhancement of Proton Conductivity in Perfluorosulfonic Acid Membranes with the Addition of Hydrophilic Fillers
Source: ACS Omega. 2026 Aug 4;11(32):47374–80. doi: 10.1021/acsomega.6c00896 (PMC13491494; doi:10.1021/acsomega.6c00896)
Supplement: Supplementary file 1 [file ao6c00896_si_001.pdf]

Supporting Information

Enhancement of Proton Conductivity in Perfluorosulfonic Acid Membranes with Addition of Hydrophilic Fillers

Tatsuki Yasuda[1, 2], Akihito Yamano[2], Ryuji Ohno[1,2] and Katsuyoshi Kakinuma[1, 2, 3]

[1]Integrated Graduate School of Medicine, Engineering, and Agricultural Sciences, University of Yamanashi, Takeda 4, Kofu, Yamanashi, 400-8511, Japan

[2]Hydrogen and Fuel Cell Nanomaterials Center, Clean Energy Research Center, University of Yamanashi, Miyamae 6-43, Kofu, 400-0021, Japan

[3]Clean Energy Research Center, University of Yamanashi, Takeda 4, Kofu, 400-8511, Japan

Corresponding Author: Katsuyoshi Kakinuma, kkakinuma@yamanashi.ac.jp

*Corresponding Author

Tel/Fax: +81-55-254-7143; E-mail: kkakinuma@yamanashi.ac.jp

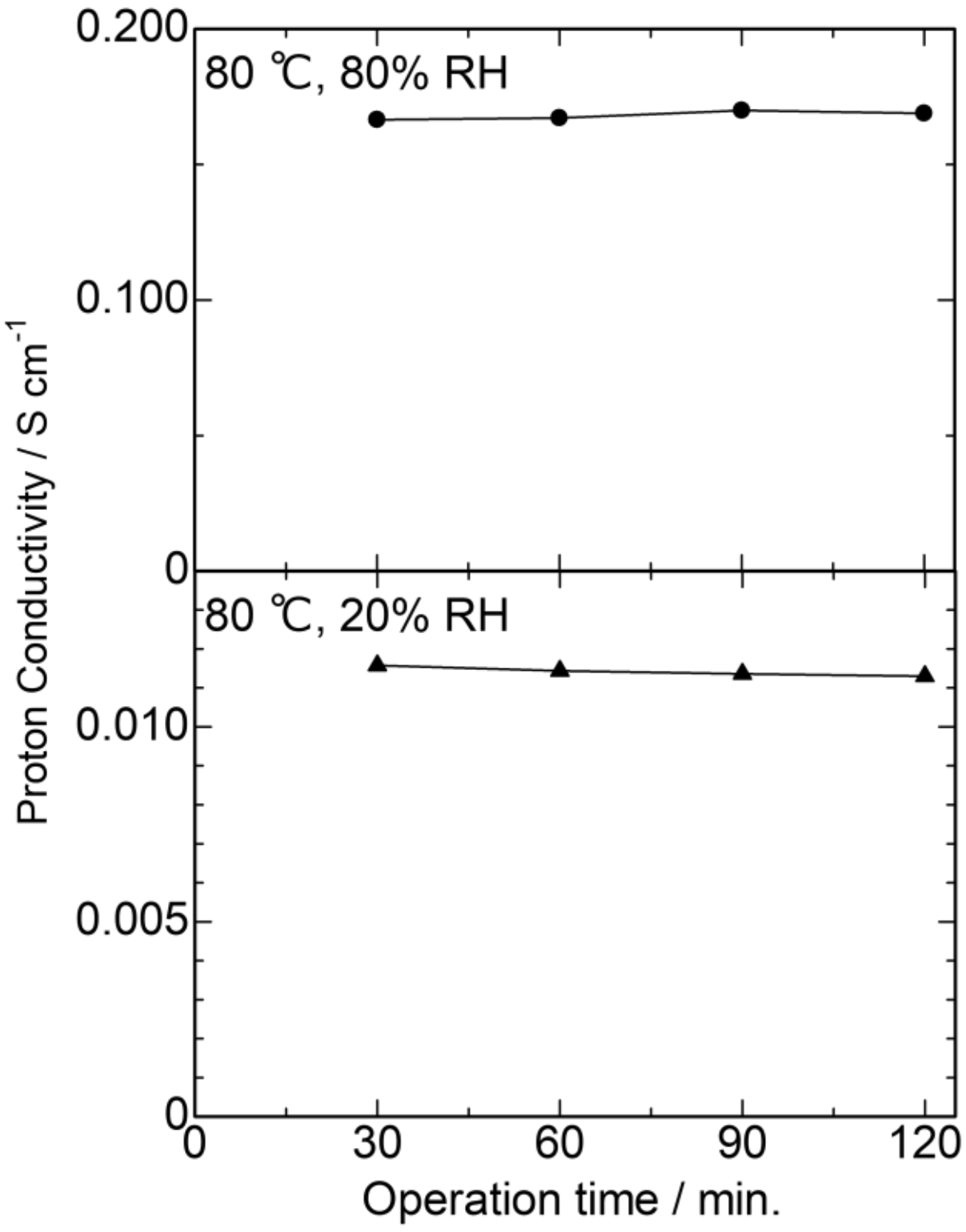

Fig. S1 5 wt.% CMCNF/Nafion electrolyte membrane steady state proton conductivity.
